# Self-reported occupational exposure to HIV and factors influencing its management practice: a study of healthcare workers in Tumbi and Dodoma Hospitals, Tanzania

**DOI:** 10.1186/1472-6963-13-276

**Published:** 2013-07-17

**Authors:** Kijakazi O Mashoto, Godfrey M Mubyazi, Hussein Mohamed, Hamisi M Malebo

**Affiliations:** 1 National Institute for Medical Research, P.O.Box 9653, Dar es Salaam, Tanzania; 2 Muhimbili University of Health and Allied Sciences, P.O.Box 65000, Dar es Salaam, Tanzania

**Keywords:** HIV, Occupational exposure, Healthcare workers

## Abstract

**Background:**

Blood borne infectious agents such as hepatitis B virus (HBV), hepatitis C virus (HCV) and human immune deficiency virus (HIV) constitute a major occupational hazard for healthcare workers (HCWs). To some degree it is inevitable that HCWs sustain injuries from sharp objects such as needles, scalpels and splintered bone during execution of their duties. However, in Tanzania, there is little or no information on factors that influence the practice of managing occupational exposure to HIV by HCWs. This study was conducted to determine the prevalence of self-reported occupational exposure to HIV among HCWs and explore factors that influence the practice of managing occupational exposure to HIV by HCWs in Tanzania.

**Methods:**

Self-administered questionnaire was designed to gather information of healthcare workers’ occupational exposures in the past 12 months and circumstances in which these injuries occurred. Practice of managing occupational exposure was assessed by the following questions:

**Results:**

Nearly half of the HCWs had experienced at least one occupational injury in the past 12 months. Though most of the occupational exposures to HIV were experienced by female nurses, non-medical hospital staff received PEP more frequently than nurses and doctors. Doctors and nurses frequently encountered occupational injuries in surgery room and labor room respectively. HCWs with knowledge on the possibility of HIV transmission and those who knew whom to contact in event of occupational exposure to HIV were less likely to have poor practice of managing occupational exposure.

**Conclusion:**

Needle stick injuries and splashes are common among HCWs at Tumbi and Dodoma hospitals. Knowledge of the risk of HIV transmission due to occupational exposure and knowing whom to contact in event of exposure predicted practice of managing the exposure. Thus provision of health education on occupational exposure may strengthen healthcare workers’ practices to manage occupational exposure.

## Background

Blood borne virus (BBV) infection has long been recognized as an important hazard for patients and healthcare workers (HCW) since the 1960s [1]. BBVs are viruses that are carried in blood of some people and they are potential for causing severe disease in certain people and few or no symptoms in others. The virus spreads to another person, whether the carrier of the virus is ill or not. The main BBVs of concern are hepatitis B virus (HBV), hepatitis C virus (HCV) and hepatitis D virus (HDV), which all cause hepatitis, a disease of the liver; whereas the human immunodeficiency virus (HIV) causes the acquired immune deficiency syndrome (AIDS), affecting the immune system. BBVs can also be found in body fluids other than blood, such as, semen, vaginal secretions and breast milk. BBVs constitute a major occupational hazard for healthcare workers (HCW), especially in highly resource constrained countries [2].

To some degree it is inevitable that HCWs sustain injuries from sharp objects such as needles, scalpels and splintered bone during execution of their healthcare duties. In addition, HCW’s mucosa may be exposed to droplets or splashes of blood, saliva and urine. Patients showing erratic behavior may inflict bite and scratch wounds. These incidents, herein being referred to as occupational exposure, carry the risk of transmission of infectious agents of which HIV, HBV and HCV are the most relevant [3]. In the context of hospital settings, the most common exposures are needle-stick injuries and splashes with body fluids, blood being most potent of all [4-6]. As the prevalence of the HIV infection continues to rise, HCWs in all geographic regions can expect an increasing frequency in the number or incidences of contacts to patients with HIV/AIDS.

Accidental injuries of both percutaneous and muco-cutaneous types are quite common as shown by studies conducted in developed and developing countries [4,7-9]. Sub-Saharan Africa has the highest prevalence of HIV-infected patients and the highest incidences of occupational exposures [10-12]. Therefore, preventive measures and response to blood exposure accidents are necessary to mitigate the risk of exposure.

Despite following ‘universal precautions’, accidental exposure may occur while performing invasive procedures and handling high risk fluids [12,13]. Studies have extensively reported suboptimal and non-uniform adherence to standard precautions by HCWs in developing countries as in developed ones [12,14,15]. It is evident the occurrence of percutaneous injury and muco-cutaneous blood exposure is inversely related to routine standard precaution compliance [8].

Avoiding occupational blood exposures by adhering to universal precautions and post exposure management are integral components of a complete program to prevent HIV infection following occupational exposure and are important elements of workplace safety. There are two main strategies for managing occupational exposure to blood. The first approach is to provide empirical treatment with two or more antiretroviral drugs unless additional information (e.g., the result of an HIV test in the source patient or a detailed description of the exposure) suggests that this treatment is not warranted. The second approach is to conduct a thorough assessment of the exposure (including an HIV test in the source patient if HIV infection has not already been diagnosed) and then initiate antiretroviral treatment only if the exposure poses a risk of HIV transmission. However, this will only be practical if exposed HCWs report the event.

The prescription of antiretroviral therapy as post-exposure prophylaxis (PEP) following significant potential exposure to HIV has now become routine.Thus, it is important that individuals with potential risk of exposure are aware of the procedures to follow and know where their first point of contact should be if an incident occurs. PEP is a medical response to prevent transmission of pathogens after potential exposure and refers to comprehensive management instituted to minimize the risk of infection following potential exposure to HIV. This includes first aid, counseling, risk assessment, relevant laboratory investigations based on the informed consent of the exposed person and source and depending on the risk assessment, the provision of short term (28 days) of antiretroviral drugs, along with follow-up evaluation [16,17]. PEP for HIV exposure is best when started within golden period of <2 hours and there is little benefit after 72 hours. Knowledge on the exposure mechanisms, transmission risks and prevention methods could assist hospital staff and managers to create a safe working environment free of unnecessary fear or anxiety [18]. In Tanzania, there is no information on factors that influence the practice of managing occupational exposure to HIV by HCWs. Therefore, the objective of this study was to determine the prevalence of self-reported occupational exposure to HIV among HCWs and explore factors that influence the practice of managing occupational exposures by HCWs in Tanzania.

## Methods

### Study settings

This study was conducted in two regions located far away from each other in Tanzania. In each region, it covered Regional Hospitals, namely, Tumbi Regional Hospital in Coast region near Dar es Salaam and Dodoma Regional Hospital in Dodoma Region. Besides the routine and referral services given to the clients from within and beyond their catchment areas, the proposed Tumbi hospital is potentially characterized with higher chances of exposure to occupational hazards due to the fact that it is located in an area with relatively higher levels of road accidents, serving people from different regions in Tanzania who face accidents on their way to or from Dar es Salaam. Dodoma hospital was selected to represent other hospitals which do not receive a lot of road accident cases.

### Study design and participants

The study was cross-sectional design which involved HCWs based at Tumbi and Dodoma Hospitals. HCWs included were medical and dental specialists, medical and dental officers, assistant medical and dental officers, medical and dental assistants, clinical officers, assistant clinical officers, laboratory technicians and technologists, radiologists, radiographers, physiotherapists, nurses and health attendants.

### Sample size and sampling

The sample size for the quantitative survey was determined using the following formula n = Z^2^ p (1-p)/d^2^ where, **Z**_***α***_ = value at a specified confidence level, **P** = approximate proportion of the event in the population and **d** = acceptable margin of error in estimating the true population proportion [19]. The proportion of Tanzanian HCWs who encounter needle-stick injuries is 53% [6] and given 1.96 value of the 95% confidence interval and 0.04 an acceptable margin of error, the minimum sample size required for the study was 598. Due to low number of HCWs employed at the selected hospital, all HCWs in the two hospitals were invited to participate in the study. However, only 401 HCWs completed the questionnaire.

### Data collection

The questionnaire was developed in English and translated in Kiswahili and then back translated in English. Two weeks before data collection, 10% of the questionnaires (40) were pre-tested in Mwananyamala hospital. Self-administered questionnaire was designed to gather information of healthcare workers’ occupational exposures in the past 12 months and circumstances in which these injuries occurred. Practice of managing occupational exposure was assessed by asking HCWs if they tested for HIV after occupational exposure, if they knew the patient’s/source’s HIV status, and if they reported the exposure to the focal person and was counseled. Other variables collected were participant’s sex, age, place of work, level of education/professional training attained, and year of employment in the current work (aimed to assess their duration and experience at workplace).

During data collection, four researchers were stationed at each hospital. HCWs were asked to return filled questionnaires to these researchers. The returned questionnaires were checked for completeness, in case of incompleteness, the questionnaire was given back to the respondent and asked to complete the questionnaire.

### Ethical considerations

Ethical clearance was obtained from the Medical Research Coordinating Committee (MRCC) through National Institute for Medical Research (NIMR) Secretariat. Regional and District/Municipal Government and Health Authorities for Pwani and Dodoma Regions also gave permission for this study to be undertaken at stated Hospitals. The final decision to participate in the study was taken by the HCWs after reading the consent form.

### Data analysis and statistical techniques

Data were entered in the computer using Epi-info program and the analysis was performed using SPSS version 17. The response options were 0 = No and 1 = Yes for the following questions: “have you tested for HIV after occupational exposure?”, “did you know the patient’s/source’s HIV status?”, and “did you report the exposure to the focal person?” Thus the score for managing occupational exposure was then obtained by summing up the three questions. The higher the score the good the post exposure management practice. For purpose of analysis the score was dichotomized on a median split into 0 = poor practice and 1 = good practice.

The response options were 0 = never, 1 = once, 2 = two to five times and 3 = more than five times for the following questions: “In the past 12 months, how frequently have you experienced needle-stick injury?” “In the past 12 months, how frequently have you been wounded with a blood contaminated sharp object?” “In the past 12 months, how frequently have you been splashed by blood or any body fluids?” “In the past 12 months, how frequently have you experienced non intact skin exposure (exposed skin that is chapped, abraded, or afflicted with dermatitis come into contact with blood or body fluids)?” and “In the past 12 months, how frequently have you experienced human bite injury (bites resulting in blood exposure to either person involved)?”. Summation of the five items gave the score for occupational exposure to HIV which was then dichotomized into never exposed and encountered at least one type of occupational exposure to HIV.

The proportions of HCWs with poor or good practices were compared by means of Chi-squared tests after study participants were categorized into those with poor and good practice of managing occupational exposure to HIV infection. Since the prevalence of poor practice of managing occupational exposure was higher than 10%, generalized linear regression models were applied in attempt to perform multivariate analysis. In this case, the log-binomial regression analyses were performed with statistical significance checked on the basis of p-value of ≤ 0.05 at 95% confidence intervals (CI) for the corresponding prevalence ratios (PRs). All the independent variables entered in the log binomial regression analysis were significantly associated with the dependent variable (managing occupational exposure to HIV) at bivariate analysis.

## Results

### Response rate and sample characteristics

A total of 401 HCWs participated in the survey at both study Hospitals. This led to an overall response rate of 67%. Non respondents were individuals who were absent during the survey days/time. Most of the participants were female nurses, and these accounted to 83% of all interviewees.

### Prevalence and types of occupational exposure to HIV

Nearly half of the HCWs had experienced at least one occupational injury in the past 12 months. The prevalence of occupational exposure to HIV varied significantly with type of cadre. Doctors and nurses had high prevalence than other hospital staffs and in most cases doctors knew the HIV status of the source (Table 1).

**Table 1:** Exposed healthcare workers (n = 192)

	**Doctors (%)**	**Nurses (%)**	**Others (%)**	**All (%)**
Exposed HCW	47 (24.5)	92 (47.9)	53 (27.6)	192 (47.9)
Male	30 (47.6)	17 (27.0)	16 (25.4)	63 (32.8)
Female	17 (13.2)	75 (58.1)**	36 (67.9)	128 (66.6)
Tested for HIV	29 (61.7)	56 (60.9)	36 (67.9)	121 (63.0)
Source status known	39 (83.0)**	64 (69.9)	29 (54.7)	132 (68.8)
Received PEP	12 (25.5)	24 (26.1)	25 (47.2)*	61 (31.8)
PEP use as instructed	8 (66.7)	17 (65.4)	16 (69.6)	41 (67.2)

**p < 0.0001; *p < 0.005.

HCWs in Tumbi hospital had high prevalence of occupational exposure to HIV compared to HCWs in Dodoma hospital. The most frequently reported type of occupational exposure was blood or body fluid splash and the least frequently reported type of occupational exposure to HIV was human bite (Table 2). Most of the occupational injuries were encountered by doctors and nurses in surgery room and labor room respectively (Table 3).

**Table 2:** Management and types of occupational exposure to HIV in the past 12 months by hospital (n = 401)

	**Tumbi hospital (%)**	**Dodoma hospital (%)**	**All (%)**
**Occupational injury**			
Needle stick (used needle)	64 (37.4)*	41 (17.8)	105 (26.2)
Blood stained sharp objects	64 (37.4)*	37 (16.1)	101 (25.2)
Blood or body fluid splash	81 (47.4)*	73 (31.7)	154 (38.4)
Blood or body fluid contact with ulcerated or abraded skin	63 (36.8)*	38 (16.5)	101 (25.2)
Human bite	45 (26.3)*	21 (9.1)	66 (16.5)
At least one occupational injury	98 (57.3)*	94 (40.9)	192 (47.9)
**Managing occupational exposure**			
Good practice	118 (69.0)	158 (68.7)	276 (68.8)

*p < 0.001.

**Table 3:** Circumstance and sections in which occupational exposure occurred (n = 192)

	**Doctors (%)**	**Nurses, (%)**	**Others (%)**	**All (%)**
Surgery room	29 (61.7)**	9 (9.8)	5 (9.4)	43 (22.4)
When cleaning	4 (8.5)	18 (19.6)	11 (20.8)	33 (17.2)
Laundry	4 (8.5)	6 (6.5)	9 (17.0)	19 (9.9)
When injecting	7 (14.9)	27 (29.3)*	7 (13.2)	41 (21.4)
Labour ward	15 (31.9)	26 (28.3)	11 (20.8)	52 (27.1)
Emergency room	5 (10.6)	12 (13.0)	3 (5.7)	20 (10.4)
Dressing room	2 (4.3)	6 (6.5)	5 (9.4)	13 (6.8)

**p < 0.0001; *p < 0.005.

### Practice of managing occupational exposure to HIV

Good practice of managing occupational exposure to HIV was reported in 68.8% of the HCW. In bivariate analysis age group, sex, knowing whom to contact in the event of occupational exposure and possibility of HIV transmission at work place associated statistically significantly with practice of managing occupational exposure to HIV. Knowing whom to contact in event of occupational exposure, older HCWs, higher proportion of females and those knowing that HIV can be transmitted through occupational exposure had good practice of managing occupational exposure than their counterparts. However, in multivariate analysis only knowledge on possibility of HIV transmission at workplace and knowing whom to contact in event of occupational exposure maintained their significant association with practice of managing occupational exposure. HCWs with knowledge on the possibility of HIV transmission and those who knew whom to contact in event of occupational exposure to HIV were less likely to have poor practice of managing occupational exposure (Table 4). Results remain unchanged even after stratifying analysis by hospital.

**Table 4:** Predictors of poor practice of managing occupational exposure to HIV

	**OR**	**95% CI**
**Sex**		
Male	1.4	0.9 – 1.9
Female	1	
**Age**		
< 30 years	1.6	0.8 – 2.9
30 – 50 years	1.4	0.8 – 2.6
>50 years	1	
**HIV transmission possibility at work place**		
Yes	0.5	0.2 – 0.9**
No	1	
**Know whom to contact in event of exposure**		
Yes	0.6	0.4 – 0.7*
No	1	

*p < 0.0001; **p < 0.05.

## Discussion

Although the prevalence of needle stick injuries in the present study is low compared to the study conducted in Mwanza and Dar es Salaam, Tanzania [9,20], HCWs in the study hospitals were still at risk of contracting HIV infections through needle stick injuries and splashes which were common in this study. In overall, labor room was the most risky area across the groups of workers in this study. Surgical procedures are by far the most risky for doctors. Apart from labor ward, injecting and cleaning are the most risky tasks for the nurses. This is consistent with reports documented elsewhere, identifying the emergency room, surgery, or gynecology department as having the highest risk of occupational exposure [21]. Meanwhile, it can be seen that apart from the labor ward, injecting and cleaning practices were found as being the most risky tasks for nurses. Doctors and nurses more frequently reported the occupational exposure than other cadres of the hospital personnel. It is worth noting that other researchers reported that nurses were more frequently than doctors, interns, and medical students exposed to the risk of HIV infection [9,21-23].

Tumbi Hospital had high prevalence of occupational exposure than the Dodoma Hospital. However, Tumbi hospital receives many emergency cases due to road accidents and more often the finds workers unprepared and without enough protective gears. HCWs based in a busy hospital are usually extremely hectic due to pressure of work and heavy workload thus ensuring personal protection may not always be a priority (or feasible thing) to them. Thus the risk of HIV transmission may be increased as HCWs frequently encounter occupational exposure to HIV. To minimize this risk of HIV transmission in hospital setting, the importance of adhering to universal precautions in whatever situation should be emphasized.

One third of the studied HCWs did not report the event of occupational exposure they encountered. Our findings may be compared to findings of a study conducted in three teaching hospitals of the United State of America, which revealed that only 30% of the needle stick injuries recalled by subjects were reported. However, it is important to note that the United State of America study reported the proportion of HCWs who encountered needle stick injury only while we report the proportion of HCWs who encountered any type of occupational exposure Lack of knowledge about the reporting mechanism is one of the principal reasons for not reporting the event of occupational exposure [24]. Thus a person to be contacted in the event of occupational exposure should be known by all HCWs at hospital level.

Health care workers who recognize the presence of occupational HIV risk are apt to be motivated to practice universal infection control precautions [25]. The same have been observed in this study. HCWs with knowledge on the risk of HIV transmission at hospital setting had good practice of managing occupational exposure in terms of making sure that they undertake appropriate post exposure measures. In order to strengthen HCWs’ practice of managing occupational exposure to HIV infections, in-house training on regular basis is recommended.

This study relied on self-reported data in estimating the prevalence of occupational exposure to HIV. A common threat to the validity of self-reports that can lead to information bias is social desirability and recall bias. There is a possibility that socially desired and undesired behaviors have been over- and under-estimated in this study, respectively.

## Conclusion

Indeed, HCWs at Tumbi and Dodoma hospitals are at high risk of HIV infection due to needle stick injuries and splashes. However, nurses have higher risk of exposure compared to other HCWs, and this calls for specific interventions to target this particular group.

HCWs preparedness to accidents in Tanzania is paramount to hospitals which have many reported accidents throughout the year and efforts should be made to put in place disaster preparedness tools to mitigate the effects of occupational health exposure to HIV especially in Tumbi hospital which receives many patients from accidents throughout a year. Knowledge of the risk of HIV transmission due to occupational exposure and whom to contact in the event of occupational exposure seems to influence practices of HCWs in relation to management of occupational exposure. Thus, there is need for provision and strengthening of health education on occupational exposure to HIV among HCWs.

## Abbreviations

AIDS: Acquired immunodeficiency syndrome; BBV: Blood borne virus; HBV: Hepatitis B virus; HCV: Hepatitis C virus; HCWs: Health care workers; HIV: Human immunodeficiency virus; MoHSW: Ministry of health and social welfare; MRCC: Medical research coordinating committee; NIMR: National institute for medical research; PEP: Post exposure prophylaxis; PPE: Person protective equipment; TANHER: Tanzania health research forum; WHO: World health organization.

## Competing interests

The authors declare that they have no competing interests.

## Authors’ contributions

KOM: Principal investigator, conceived of the study, designed the study, collected data, statistical analysis and manuscript writing. GMM: designed study and manuscript writing. HM: Participated in design of study and manuscript writing. HMM: commented on the paper and provided valuable guidance for manuscript write up. All authors read and approved the final manuscript.

## Pre-publication history

The pre-publication history for this paper can be accessed here:

http://www.biomedcentral.com/1472-6963/13/276/prepub

## References

[B1] Strader D Wright T Thomas D Seef L Diagnosis, management and treatment of Hepatisis C Hepatology 2004 39 1147 1171 10.1002/hep.20119 15057920

[B2] Merchant RC Mayer KH Becker BM Delong AK Hogan JW Predictors of the initiation of HIV post exposure prophylaxis in Rhode Island emergency departments AIDS Patient Care STDS 2008 22 1 41 52 10.1089/apc.2007.0031 18095841 PMC3206608

[B3] Frijstein G Hortensius J Zaaijer HL Needle- stick injuries and infectious patients in a major academic medical centre from 2003 to 2010 Neth J Med 2010 69 10 465 468 22058270

[B4] Nsubuga FM Jaakkola MS Needle sticks injuries among nurses in sub-Saharan Africa Trop Med Int Health 2005 10 773 781 10.1111/j.1365-3156.2005.01453.x 16045464

[B5] Denise C Anne B Laptey PR, Gayle HD Transmission of HIV in health care settings HIV/AIDS prevention and care in resource constrained settings: A Handbook for the Development and Management of Programs 2001 Family Health International 501 516

[B6] Manyele SV Ngonyani HAM Eliakimu E The status of occupational safety among health service providers in hospitals in Tanzania Tanzan J Health Res 2008 10 3 133 140 10.4314/thrb.v10i3.14356 19024341

[B7] Shariati B Shahidzadeh-Mahani A Oveysi T Akhlaghi H Accidental exposure to blood in medical interns of Tehran University of Medical Sciences Journal of occupational health [Internet] 2007 49 4 317 321 Available from: http://www.ncbi.nlm.nih.gov/pubmed/17690526 10.1539/joh.49.317 17690526

[B8] Doebbeling B Vaughn TE McCoy KD Percutaneous injury, blood exposure and adherence to standard precautions: are hospital based healthcare providers still at risk? Clin Infect Dis 2003 37 1006 1013 10.1086/377535 14523763

[B9] Gumodoka B Favot I Berege Z Dolmans WM Occupational exposure to the risk of HIV infection among health care workers in Mwanza Region, United Republic of Tanzania Bulletin WHO 1997 75 2 133 140 PMC2486935 9185365

[B10] Sagoe-Moses C Risk to healthcare workers in developing countries N Engl J Med 2001 345 7 538 541 10.1056/NEJM200108163450711 11519511

[B11] Hutin Y Hauri A Chiarello L Catlin M Stilwell B Best infection control practices for intradermal, subcutaneous, and intramuscular needle injections Bulletin WHO 2003 81 2 491 500 PMC2572501 12973641

[B12] WHO The World Health Report: reducing risks, promoting healthy life 2002 Geneva, Switzerland: World Health Organization

[B13] MMWR Surveillance for occupationally acquired HIV infection 1992 United States, 1981–1992 1406579

[B14] Bennet G Mansell I Universal precautions: a survey of community nurses’ experience and practice J Clin Nurs 2003 13 4 413 421 10.1046/j.1365-2702.2003.00889.x 15086627

[B15] Zhang M Wang H Miao J Du X Li T Occupational exposure to blood and body fluids among healthcare workers in a general hospital, China Am J Ind Med 2009 52 2 89 98 10.1002/ajim.20645 19016263

[B16] Sharma A Marfatiya YA Ghiya R Post-exposure prophylaxis for HIV Indian J Sex Trans Dis and AIDS 2007 28 2

[B17] Government of India Management of occupational exposure including post exposure prophylaxis for HIV 2009 New Delhi: NACO, Ministry of Health and Family Welfare

[B18] Twitchell K Bloodborne pathogens: What you need to know - Part I AAOHN J 2003 51 38–45 46 12596344

[B19] Lwanga SK Lemeshow S Sample size determination in health studies: A practical manual 1991 Geneva: World Health Organization

[B20] Chagani MM Manji KP Manji MP Sheriff FG Healthcare workers’ knowledge, attitudes and practice on post exposure prophylaxis for HIV in Dar es Salaam Tanzania Medical Journal 2007 25 2 1 6

[B21] Lin C Li L Zunyou W Sheng W Manhong J Occupational exposure to HIV among healthcare providers: a qualitative study in Yunnan China J Assoc Physicians AIDS Care 2008 7 1 35 41 10.1177/1545109707302089 PMC2791920 17641135

[B22] Pont F Hatungimana V Guiguet M Assessment of occupational exposure to human immunodeficiency virus and hepatitis C virus in a referral hospital in Burundi, Central Africa Infect Control Hosp Epidemiol 2003 24 10 717 718 10.1086/502908 14587928

[B23] Karstaedt A Pantanowitz L Occupational exposure of interns to blood in an area of high HIV seroprevalence S Afr Med J 2001 91 57 61 11236300

[B24] Mangione CM Gerberding JL Cummings SR Occupational exposure to HIV: frequency and rates of underreporting of percutaneous and muco-cutaneous exposures by medical house staff Am J Med 1991 90 1 85 90 10.1016/0002-9343(91)90510-5 1986594

[B25] Gerberding JL Does knowledge of human immunodeficiency virus infection decrease the frequency of occupational exposure to blood? Am J Med 1991 91 3 Supplement 2 S308 S311 10.1016/0002-9343(91)90387-d 1928184

